# Metabolic Syndrome Rather Than Other Phenotypes in PCOS as a Predictive Indicator for Clinical Outcomes in IVF: Comprehensive Phenotypic Assessment across All PCOS Classifications

**DOI:** 10.3390/jcm12155073

**Published:** 2023-08-02

**Authors:** Manfei Si, Wanxue Xu, Xinyu Qi, Huahua Jiang, Yue Zhao, Rong Li, Xiaoyu Long, Jie Qiao

**Affiliations:** 1 Center for Reproductive Medicine, Department of Obstetrics and Gynecology, Peking University Third Hospital, Beijing 100191, China; 2 National Clinical Research Center for Obstetrics and Gynecology, Peking University Third Hospital, Beijing 100191, China; 3 Key Laboratory of Assisted Reproduction, Peking University, Ministry of Education, Beijing 100191, China; 4 Beijing Key Laboratory of Reproductive Endocrinology and Assisted Reproductive Technology, Beijing 100191, China; 5 Beijing Advanced Innovation Center for Genomics, Beijing 100191, China; 6 Peking-Tsinghua Center for Life Sciences, Peking University, Beijing 100191, China

**Keywords:** metabolic syndrome, polycystic ovary syndrome, phenotypes, in vitro fertilization, clinical outcomes

## Abstract

Polycystic ovary syndrome (PCOS) is a well-recognized, multi-system metabolic disorder affecting fertility. Although various classification methods have been proposed to assess the phenotypic heterogeneity of PCOS, there is currently no reliable phenotype for predicting clinical IVF outcomes. This retrospective study, as a comprehensive phenotypic assessment across all PCOS classifications, aimed to identify dependable phenotypes that can serve as predictors for IVF and pregnancy outcomes. The study included 1313 PCOS patients who received their initial IVF treatment between January 2019 and December 2021. The phenotypes reflect the diverse metabolic and hormonal characteristics in this study. Phenotype A, within the Rotterdam criteria classification, exhibited the highest anti-Müllerian hormone levels (AMH), while phenotype D displayed the lowest Homeostasis Model Assessment of Insulin Resistance (HOMA-IR) values. Both the hyperandrogenism (HA) phenotype within HA-based classification and the overweight phenotype within the body-mass-index-based classification showed increased HOMA-IR and metabolic syndrome (MetS). The MetS phenotype had higher free androgen index and a lower AMH. Notably, the MetS-based classification system demonstrated an independent association of MetS with cumulative live birth, preterm birth, and gestational diabetes mellitus as a contributing risk factor for PCOS patients undergoing IVF (*p* < 0.05). These findings carry noteworthy implications for advancing clinical management strategies for PCOS.

## 1. Introduction

Polycystic ovary syndrome (PCOS) is widely acknowledged as one of the most prevalent endocrine and metabolic disorders affecting women in their reproductive years [1]. PCOS is characterized by the presence of hyperandrogenism (HA) and ovarian dysfunction, which encompasses ovulatory dysfunction (OA) and/or polycystic ovarian morphology (PCOM) [2,3].

PCOS is known for its heterogeneous clinical manifestations, encompassing both reproductive phenotypes (OA, HA, PCOM, and excessive luteinizing hormone secretion) and metabolic phenotypes (obesity, hyperinsulinemia, insulin resistance, etc.). Additionally, insulin resistance (IR) is commonly observed in patients with PCOS, particularly in those who are obese, although some lean individuals with PCOS also exhibit IR. Hyperinsulinemia promotes insulin-like growth factor 1 (IGF-1) synthesis in ovarian interstitial cells, thereby intensifying the effect of LH on follicular membrane cells, leading to increased androgen production and reduced levels of sex-hormone-binding globulin (SHBG), ultimately resulting in elevated free testosterone levels [4,5]. Moreover, PCOS is associated with dysregulation in glucolipid metabolism, impaired glucose tolerance, such as a higher prevalence of type 2 diabetes mellitus, lower levels of high-density lipoprotein cholesterol (HDL-C), and elevated levels of triglycerides (TG) and low-density lipoprotein cholesterol (LDL-C) compared to women without the syndrome [6,7]. These factors, along with BMI and blood pressure, collectively contribute to the increased cardiometabolic risk observed in individuals with PCOS [8].

Nowadays, the Rotterdam criteria lists four phenotypes which are widely employed as the primary approach [9,10]. This classification recognizes the presence of distinct clinical phenotypes of PCOS, including a complete type of PCOS and those lacking overt manifestations of HA, OA, or PCOM features. Given the considerable variability observed in the short-term and long-term risks, along with the intricate presence of comorbidities in individuals with PCOS, a noteworthy proposition has emerged, advocating for a comprehensive evaluation of the inherent distinct phenotypes characterizing PCOS [11,12]. Thus, alternative classifications focusing on the metabolic characteristics of patients have also been proposed. These classifications take into consideration factors such as (i) the presence of overweight/obesity or central obesity (referred to as BMI-based PCOS), (ii) impaired glucose tolerance or diabetes or metabolic syndrome (MetS) (MetS-based PCOS), and (iii) classic HA (HA-based PCOS) [13].

Women with PCOS experience a notable decline in natural fertility, yet the utilization of in vitro fertilization (IVF) treatment substantially influences the reproductive outcomes of infertile patients with PCOS. Furthermore, extensive research has indicated that women diagnosed with PCOS exhibit an elevated susceptibility to pregnancy complications [14,15,16]. Nonetheless, comprehensive investigations exploring the impact of different classification approaches on IVF outcomes, obstetric outcomes, and complications in PCOS patients are still lacking.

Accurate typing and diagnosis of PCOS are pivotal in alleviating clinical symptoms, addressing fertility concerns, and improving the overall quality of life for individuals affected by PCOS. Consequently, this retrospective study aims to elucidate and compare various phenotypes of PCOS patients using different classification approaches with a specific focus on population characteristics, particularly multi-system metabolic indicators. Furthermore, the study seeks to evaluate detailed pregnancy outcomes, including the cumulative clinical pregnancy rate, cumulative live birth rate, preterm birth rate, and obstetric complications.

## 2. Materials and Methods

### 2.1. Participants

Study participants included 1313 consecutive patients diagnosed with PCOS based on the Rotterdam diagnostic criteria. These patients underwent their initial IVF treatment with GnRH-ant protocol between January 2019 and December 2021. Exclusion criteria consisted of patients below 20 or above 40 years of age, cycles with missing embryo information or clinical pregnancy data, patients suffering from chromosomal abnormalities, uterine or ovarian diseases or surgeries.

PCOS diagnosis in patients required meeting two or more of three of the following criteria: OA, clinical or biologic HA, and PCOM, while excluding other etiologies (congenital adrenal hyperplasia, Cushing’s syndrome, androgen-secreting tumors and other disorders caused anovulation such as hypothalamic and pituitary diseases) [17,18]. Ovulatory dysfunction was defined as menstrual cycles lasting less than 21 days or greater than 35 days. Biologic HA was assessed based on reliable androgen assays, with total testosterone (TT) level >2.53 nmol/L, androstenedione (AND) level >11.5 nmol/L or dehydroepiandrosterone sulfate (DHEAS) level >10.6 μmol/L. Additionally, the free androgen index (FAI) was calculated as (TT/SHBG) × 100% [19]. PCOM features were identified through transvaginal pelvic ultrasound, characterized by ovarian volume > 10 mL or more than 12 antral follicles (AFC) (2 to 9 mm in diameter) in either ovary or both [20].

According to the previous proposal by Azziz et al. [3] and the NIH consensus-panel-recommended phenotype classification, this study classified PCOS patients into four phenotypes based on Rotterdam criteria (RC-PCOS): phenotype A, characterized by OA, HA, and PCOM; phenotype B, displaying OA and HA, but no PCOM; phenotype C exhibiting HA and PCOM; and phenotype D, presenting only with OA and PCOM [21,22,23]. Additionally, these PCOS patients were also divided into different PCOS sub-phenotypes according to their BMI and the status of HA (BMI-based and HA-based classification).

### 2.2. Laboratory Tests

During days 2–4 of the menstrual cycle, serum levels of various hormones including FSH, LH, anti-Mullerian hormone (AMH), TT, AND, DHEAS, and SHBG were measured. The participants’ metabolic profiles were obtained from electronic medical records, which included measurements of blood pressure, plasma glucose, plasma insulin, total cholesterol (TC), TG, HDL-C, LDL-C and IGF-1 levels. Insulin resistance was assessed using the Homeostasis Model Assessment of Insulin Resistance (HOMA-IR) equation as follows: HOMA-IR = insulin (mIU/L) × glucose (mmol/L)/22.5 [24]. Overweight was defined according to the established guidelines for Chinese adults with a BMI  ≥  24 kg/m^2^ [25,26].

MetS was defined based on the original NCEP ATP III definition [27], considering: (1) elevated blood pressure, defined as ≥130/85 mmHg or the use of anti-hypertensive medication; (2) elevated fasting glucose level of ≥6.1 mmol/L; (3) decreased HDL-C level of <1.30 mmol/L in women or the use of lipid-lowering medication; and (4) elevated TG level of ≥1.70 mmol/L. The presence of ≥2 of these clinical measures indicated the diagnosis of MetS, as previous study stated [28,29].

### 2.3. The GnRH-Ant Protocol

All patients diagnosed with PCOS underwent their first IVF cycle using the GnRH-ant protocol. On day 2 of the menstrual cycle, patients received daily injections of gonadotropins. Subsequently, the administration of GnRH antagonist was employed in two approaches: (1) the fixed daily protocol, where the antagonist was added on days 5–7 of the gonadotropin treatment, irrespective of the follicle size; (2) the flexible daily protocol, where the antagonist was added based on the dominant follicle size, specifically when the leading follicle measured 14 to 15 mm. Throughout the treatment, transvaginal ultrasound monitoring was performed to assess the growth of ovarian follicles and adjust the FSH dose according to the ovarian response. Administration of urinary or recombinant human chorionic gonadotropin (hCG) occurred when at least three follicles reached a diameter greater than 17 mm. Patients at high risk of ovarian hyperstimulation syndrome (OHSS) were given GnRH-a trigger of 0.2 mg. Oocyte retrieval was performed using transvaginal ultrasound-guided needle aspiration 36 h after the hCG injection. Conventional IVF was utilized for the majority of patients, while intracytoplasmic sperm injection (ICSI) was conducted specifically for couples suffering male infertility issues such as severe oligospermia, asthenospermia, teratospermia, azoospermia, anejaculation, and cases where fertilization failures occurred with conventional IVF. Half-ICSI was used as an alternative technique for patients presenting suspected fertilization difficulties.

### 2.4. Measurement of Outcomes

The assessment of IVF outcomes included the number of retrieved oocytes, maturation rate (within ICSI), fertilization rate and the rates of two pronuclei (PN) and good quality embryos. The determination of the oocyte maturation rate (only observed in ICSI) was based on the calculation of the ratio between mature MII oocytes and the total number of retrieved oocytes. The fertilization rate in IVF cycles was defined as the ratio of oocytes with one or two or multiple PN to the total number of retrieved oocytes. However, for ICSI cycles, the fertilization rate was determined by dividing the number of oocytes with one or two or multiple PN by the total number of mature MII oocytes. The 2PN rate refers to normal fertilization rate, representing the percentage of two PN out of the total number of retrieved oocytes or mature MII oocytes in ICSI cycles. During the transfer day, the assessment of embryo grading took place. The evaluation of cleavage-stage embryos adhered to the established criteria outlined in the Istanbul Embryo Evaluation Symposium [29,30], while blastocysts were evaluated based on the Gardner grading system [31]. Embryos derived from 2PN fertilization and categorized with a score of I or II were designated as good quality. The number of good quality embryos on day 2 or 3 from the normally fertilized oocytes was calculate to determine the good quality embryo rate.

The assessment of pregnancy outcomes was examined, including cumulative clinical pregnancy rate, cumulative live birth rate, preterm birth, miscarriage, twin pregnancy, gestational diabetes mellitus (GDM), and pregnancy-induced hypertension (PIH). The definition of clinical pregnancy entailed the presence of one or more gestational sacs, as determined via transvaginal ultrasound, including normal intrauterine pregnancy, ectopic pregnancy, and simultaneous intrauterine pregnancy. Live birth was established as the occurrence of at least one newborn baby being born alive. Preterm birth was classified as delivery at <37 weeks of gestation. Miscarriage was defined as delivery at <28 weeks of gestation. GDM screening was performed at 24 weeks of gestation and involved the identification of plasma glucose levels >10 mmol/L subsequent to a 2 h oral glucose tolerance test (OGTT) with the administration of 75 g of glucose. PIH was characterized by the manifestation of hypertension, with systolic blood pressure exceeding 140 mmHg or diastolic blood pressure surpassing 90 mmHg, occurring after the 20th week of gestation, either accompanied by or without proteinuria. Cumulative clinical pregnancy was defined as the initial occurrence of pregnancy with gestational sacs after fresh embryo transfer or after cryopreserved embryo transfer within an oocyte retrieval cycle. Cumulative live birth denoted the first instance of live birth subsequent to either fresh or vitrified-warmed embryo transfer within an oocyte retrieval cycle.

### 2.5. Statistical Analysis

Statistical analyses were conducted using the IBM SPSS software (version 25.0, IBM SPSS Inc., Chicago, IL, USA). Continuous variables were checked for normality and reported as means and standard deviation (SD). Categorical variables were presented as frequencies and percentages. Group comparisons were performed using Student’s t-test or Analysis of variance (ANOVA) as appropriate for assessing differences among different phenotypes. Categorical variables were compared using Pearson’s chi-square (χ^2^) test. Logistic regression model was employed to identify independent predictors. Statistical significance was set at a *p* value of <0.05.

## 3. Results

### 3.1. The Characteristics of Patients with Different Classic Phenotypes of PCOS

**Figure 1:**
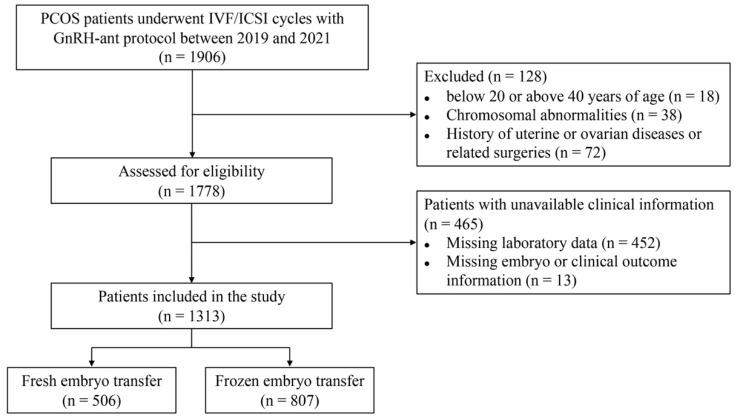
Flow chart of patient enrollment.

The study included a total of 1313 patients with PCOS who underwent IVF/ICSI cycles using the GnRH-ant protocol between January 2019 to December 2021 (Figure 1). Among these patients, 506 underwent fresh embryo transfer (ET). Based on the Rotterdam criteria, 596 women (45.4%) were diagnosed with phenotype A, 53 women (4.0%) with phenotype B, 135 women (10.3%) with phenotype C, and 529 women (40.3%) with phenotype D. The characteristics of these four PCOS types were compared and presented in Table 1. The mean age and BMI were similar among the different RC-PCOS phenotypes, as well as the fertilization rate. HA was predominantly observed in AND and DHEAS levels (*p* < 0.001), but not TT levels. Phenotype A had the highest AMH level, and there was a positive correlation between AMH levels and the number of retrieved oocytes. In contrast, phenotype D had the lowest LH/FSH ratio and HOMA-IR, and thickest endometrium on the trigger day. The prevalence of MetS was similar across the four phenotypes groups. However, a greater prevalence of MetS was observed among HA groups (phenotype A and C) compared to the non-HA group (phenotype D). Furthermore, less than half of the cases underwent fresh ET, with phenotype D displaying the highest rate of fresh ET.

As shown in Table 2, no significant difference in BMI was observed between the different PCOS phenotypes based on the presence of HA. Patients with HA had significantly higher values for LH/FSH ratio, AMH, HOMA, MetS, as well as the number of retrieved oocytes compared to patients with normal androgen level (*p* < 0.05). Consistent with the findings presented in Table 1, it was observed that patients with normal androgen level had a higher level of endometrial thickness on the trigger day.

Regarding the phenotypes of PCOS based on the presence of overweight (BMI-based PCOS), ages were similar between the two groups. Although here were no statistically significant differences in TT, AND, and DHEAS, the FAI was significantly higher in the overweight group compared to the normal-weight group. Those with PCOS and overweight exhibited metabolic disorders with higher HOMA-IR values and a higher incidence of MetS. Normal weight PCOS groups had significantly higher AMH levels, as well as a greater number of retrieved oocytes.

### 3.2. The Characteristics of Patients with or without MetS

Table 3 presents a comparison of the clinical characteristics of PCOS patients based on the presence of MetS. Among the 1313 patients, 796 (39.4%) had MetS. There were no significant differences in age, LH/FSH ratio, and levels of androgens including TT, AND, and DHEAS between MetS and non-MetS groups of PCOS patients. However, the MetS subgroup had significantly higher BMIs, FAIs, and HOMA-IR values, and lower SHBG, AMH and IGF-1 levels compared to the non-MetS group. Furthermore, the comparison revealed that the MetS group had a lower number of retrieved oocytes. Notably, this group also displayed a significantly higher rates of fresh ET (*p* < 0.001).

### 3.3. Pregnancy Outcomes in Women with Different Phenotypes of PCOS

Subsequent investigations aimed to assess the impact of different classifications on pregnancy outcomes and are presented in Table 4. Within the HA-based classification, no notable distinctions were observed in cumulative clinical pregnancy, cumulative live birth, preterm birth, miscarriage, and obstetric complications including twin pregnancy, GDM, and PIH between HA and normal HA subgroups. However, significant differences were found among phenotypes classified according to the RC-PCOS, specifically in relation to preterm birth (*p* = 0.014). Additionally, it was observed that overweight individuals with PCOS exhibited lower rates of cumulative clinical pregnancy and cumulative live birth and higher rate of miscarriage when compared to those with normal weight PCOS. Importantly, phenotypes identified within the MetS-based classification demonstrated significant differences in clinical outcomes, including cumulative clinical pregnancy, cumulative live birth, preterm birth, and GDM (*p* < 0.05), thereby indicating that the MetS-based classification exhibited superior performance in discerning obstetric outcomes.

Supplementary Table S1 showed pregnancy outcomes in fresh transfer cycles. PCOS phenotypes in MetS-classification also had significant differences in clinical pregnancy rate and live birth rate (*p* < 0.05). These findings suggest that PCOS phenotypes based on MetS can better predict the pregnancy outcomes in PCOS patients undergoing IVF/ICSI cycles with the GnRH-ant protocol.

### 3.4. Factors Associated with Pregnancy Outcomes in Women with PCOS

A logistic regression model was utilized to investigate the factors associated with cumulative pregnancy outcomes in women with PCOS (Table 5). The analysis involved adjusting for age, type of infertility, infertility duration, BMI, LH/FSH, AMH, and HOMA-IR. MetS was found to be negatively associated with cumulative live birth (*p* = 0.024), and positively associated with preterm birth and GDM (*p* < 0.05).

BMI emerged as a significant independent factor for predicting cumulative clinical pregnancy and cumulative live birth. Furthermore, the duration of infertility appeared to be related to PIH. In the context of fresh transfer cycles, MetS was identified as an independent risk factor for predicting clinical pregnancy and preterm birth (Supplementary Table S2).

## 4. Discussion

This study investigated and assessed the impact of various classifications of PCOS on endocrine and metabolic characteristics, as well as clinical IVF outcomes and obstetric complications. The findings of this study revealed a noteworthy inverse association between MetS-based classification and cumulative live birth, clinical pregnancy, preterm birth, and GDM compared to other classifications. These results underscore the significance of adopting metabolic syndrome as a classification system, as it effectively highlights the relationship between patients’ metabolic status and clinical prognosis.

Diagnostic classifications have been refined over time to better comprehend the intricate pathology and diverse clinical symptoms of PCOS. Various classification approaches have demonstrated their effectiveness in distinguishing different subgroups of PCOS, characterized by distinct metabolic, endocrine, and reproductive profiles, including HA, hormonal, and the associated metabolic disruption [20]. In the present study, it was observed that AMH levels were significantly higher in phenotype A (9.25 ± 4.98 ng/mL) compared to other phenotypes within the RC-PCOS group, while phenotype B exhibited the lowest AMH level (6.88 ± 3.88 ng/mL), potentially indicating the presence of OA and PCOM. These findings are consistent with previous research that suggests AMH serves as a marker of PCOS severity [32,33].

HA can manifest through clinical symptoms (e.g., hirsutism and acne), laboratory indicators, or a combination of both. Interestingly, within the scope of the current investigation, distinct classifications displayed varying degrees of performance in effectively discerning the biomarkers associated with HA. Soyman et al. reported the highest level of free testosterone in phenotype A and the lowest level in phenotype D [33,34], whereas Gursu et al. found that DHEAS levels were highest in phenotype A and the lowest in phenotype B [35]. Given the limitations of measuring TT and direct free testosterone in females [4,36,37], FAI was also employed in the present study. The findings have shown that phenotype C had the highest rate of high FAI among RC-PCOS phenotypes, followed by phenotype A, phenotype B, and phenotype D, which differs from a previous study [38]. This difference may be attributed to variations in the prevalence of the four phenotypes. Specifically, the present study has reported the following proportions: phenotype A (45.4%), phenotype B (4.0%), phenotype C (10.3%), and phenotype D (40.3%), whereas the previous study reported proportions of 51.9%, 23.1%, 13.1%, and 11.9%, respectively [37,38]. Furthermore, the study demonstrated that the metabolic risk associated with the ovulatory phenotype C and complete phenotype A was higher compared to the non-hyperandrogenic phenotype D, indicating that the Rotterdam classification may distribute the metabolic characteristics within the population. Although statistical significance was not observed between phenotypes B and D, a detectable different trend existed, potentially due to the limited sample size of phenotype B. Moreover, racial and ethnic disparities may influence the prevalence of RC-phenotypes in the population [39,40]. Notably, the ovulatory phenotype C demonstrated a relatively high likelihood of spontaneous pregnancy, potentially introducing enrollment deviations within the clinical setting.

The impact of HA on IR and its role in exacerbating PCOS development are well-established [10,41]. According to the HA-based classification, the HA group exhibited higher levels of insulin impairment, as indicated by relevant biomarkers, and a higher prevalence of MetS. As expected, HOMA-IR was more prominent in HA phenotypes within RC-PCOS, with non-HA phenotype D having the lowest HOMA-IR (2.64 ± 2.78). Meanwhile, there are discernible global variations in PCOS phenotypes among women belonging to diverse racial and ethnic backgrounds. A comprehensive systematic review, encompassing 30 studies, recently shed light on these variations [42]. The review findings indicated that South Asian, Indian, and Norwegian women diagnosed with PCOS face an increased risk of developing MetS, while Hispanic and Mexican women exhibit a heightened susceptibility to insulin resistance [42]. These findings are further supported by the cross-sectional study conducted by Chan et al., which involved over 1000 women with PCOS across multiple countries. This study revealed significant disparities in the prevalence of MetS and its individual components within distinct racial and ethnic clusters [43].

Overweight, considered a typical metabolic phenotype, is recognized to be linked with HA, ovulatory and metabolic dysfunction [44]. Prior research has revealed that women with PCOS, with a high BMI (≥23 kg/m^2^), experience more rapid hair growth and a higher level of FAI. Furthermore, BMI and PCOS showed an additive effect in elevating FAI levels [45]. Consistent with these findings, the similar trend was observed in the present study, where the overweight phenotype within BMI-based classification exhibited the highest FAI levels compared to the normal-weight phenotype. Additionally, in the present study, the overweight phenotype exhibited a severe metabolic condition characterized by a higher HOMA-IR and incidence of MetS, whereas the normal weight phenotype demonstrated an elevated level of AMH. Previous research had also indicated a significant positive correlation between HOMA-IR and BMI [46]. These findings highlight the importance of implementing lifestyle modifications among women with PCOS to enhance metabolic health, facilitate weight loss, and improve ovarian function.

The present investigations examining the correlation between different classifications and pregnancy outcomes revealed that BMI and MetS-based classifications had significant effects on cumulative clinical pregnancy rate and cumulative live birth rate, whereas these effects were not observed in RC-classification and HA-based classification. MetS classification was also significantly correlated with preterm birth and GDM. Meanwhile, in fresh cycle, only MetS classification presented a negatively effect on clinical pregnancy rate and live birth rate. Previous research has reported inconclusive and conflicting findings regarding the association between phenotypes and pregnancy outcomes [16]. Studies have demonstrated no difference in clinical pregnancy rates and multiple pregnancies among different the phenotypes within RC-classification and HA-based classification in Caucasians [47,48]. Ramezanali et.al. found that clinical pregnancy rate was higher in the phenotype D group (53.3%) than other groups (2.5%, 26.4% and 36.8% for phenotypes A, B and C, respectively), but that it did not reach a significant level [47]. De Vos et al. demonstrated that phenotype D had a higher live birth rate and cumulative clinical pregnancy rate compared to phenotypes A and C, after excluding the influence of phenotype B [49]. When compared to those without PCOS, the incidence of adverse pregnancy outcomes, such as ectopic pregnancy, miscarriage, preterm birth, and hypertensive disorders, was found to be higher in women with PCOS exhibiting phenotypes A and D [50]. HA phenotype PCOS is associated with increased rates of spontaneous abortion and pregnancy loss. This association may be attributed to the influence of HA on oocyte competence, embryonic development, and endometrial receptivity, which in turn can lead to chronic, low-grade inflammation. Previous studies have consistently demonstrated that women with PCOM have higher clinical pregnancy rates and cumulative live birth rates compared to women with PCOS, while there is a comparable number of follicles in both groups [51,52,53]. This observation suggests that women with PCOS experience a higher rate of miscarriage [52,53]. The oocytes competence of women with PCOS is significantly influenced by the specific PCOS phenotype they possess [54]. A recent study examined oocyte competence in nonobese women with PCOS [55]. The study revealed no significant differences in terms of MII oocytes per follicle and oocyte morphology, between the groups. However, it did find significantly higher rates of implantation, clinical pregnancies, and live births in the PCOS group (PCOM plus OA or HA, or both) and the PCOM population [52,55]. These findings provide evidence that oocyte competence varies across different PCOS phenotypes and associated morbidities, encompassing oocytes with reduced quality as well as those displaying an improved ability for fertilization and reproductive outcomes.

The presence of PCOS and its diagnostic features plays a crucial role in the observed heightened risk of pregnancy complications. Previous studies have reported that PCOS patients face a 3–4-fold increased risk of gestational hypertension and preeclampsia, a 3-fold increased risk of gestational diabetes, and a 2-fold increased likelihood of preterm birth [56,57]. The characteristics of PCOS may contribute to an elevated susceptibility to obstetric and neonatal complications [56,57]. Although earlier studies have suggested that maternal PCOS is associated with a heightened risk of adverse obstetric outcomes, this study is the first to propose the association between a MetS-based classification system and preterm birth and GDM. The multivariate analysis in this study also suggests that a MetS-based approach can act as an independent factor in determining poor obstetric outcomes.

The present study is advantageous due to the availability of detailed clinical data, which facilitated the investigation of population characteristics, including biochemical markers related to blood glucose and lipid profiles, as well as IVF and obstetric outcomes in the context of IVF treatment. The diagnosis of HA in this study was based on three biochemical markers: TT, DHEAS, and AND, thereby reducing the likelihood of misdiagnosis and misclassification of PCOS. However, there are certain limitations that should be acknowledged. Firstly, it should be noted that only PCOS patients undergoing antagonist regimens were included in this study. Secondly, the present study did not gather information regarding whether the patients had employed other intervention strategies, such as weight loss, lifestyle modifications, medication interventions, or prior fertility treatments, prior to commencing the IVF cycle. Finally, the definition of metabolic syndrome was not standardized, and the measurement of waist-to-hip ratio was not available in this study. Future research should investigate the performance of different phenotypes within central obesity and BMI-based classifications in PCOS patients. To enhance the reliability and generalizability of the findings, larger sample sizes and multi-center studies are warranted to provide physicians with more accurate and comprehensive information.

## 5. Conclusions

In conclusion, the diverse classification methods employed in this study have revealed disparities in the fundamental metabolic and reproductive characteristics of patients, as well as their predictive capacity for IVF clinical outcomes. Notably, the MetS-based classification system has revealed an independent association of MetS with cumulative live birth, preterm birth, and GDM, indicating MetS as a contributing risk factor for patients with PCOS undergoing IVF.

## Figures and Tables

**Table 1:** Clinical characteristics of phenotypes in PCOS based on the Rotterdam criteria.

Characteristics	Phenotype A (*n* = 596)	Phenotype B (*n* = 53)	Phenotype C (*n* = 135)	Phenotype D (*n* = 529)	*p* Value
Age (years)	30.14 ± 3.49	30.53 ± 2.99	30.19 ± 3.45	30.59 ± 3.43	0.163
BMI (kg/m^2^)	24.97 ± 3.81	24.99 ± 4.32	25.80 ± 4.40	24.86 ± 4.03	0.109
TT (nmol/L)	1.31 ± 0.64	1.18 ± 0.53	1.41 ± 0.78	1.06 ± 3.63	0.232
AND (nmol/L)	14.93 ± 4.68	13.66 ± 6.31	14.96 ± 5.97	6.66 ± 2.94	<0.001
DHEAS (μmol/L)	6.79 ± 2.91	8.11 ± 3.57	7.33 ± 3.37	5.33 ± 1.86	<0.001
SHBG (nmol/L)	32.28 ± 29.32	41.44 ± 35.64	36.52 ± 39.93	44.83 ± 46.01	0.018
FAI	5.89 ± 4.48	4.47 ± 4.14	7.25 ± 7.45	3.29 ± 2.25	<0.001
LH/FSH	1.05 ± 0.61	1.13 ± 0.91	1.09 ± 0.58	0.99 ± 0.72	0.153
AMH (ng/mL)	9.25 ± 4.98	6.88 ± 3.88	8.96 ± 4.55	7.16 ± 3.64	<0.001
IGF-1 (ng/mL)	222.66 ± 62.18	222.52 ± 66.20	224.55 ± 65.12	217.41 ± 67.74	0.536
HOMA-IR	2.94 ± 2.06	2.92 ± 1.90	3.26 ± 2.69	2.64 ± 2.78	0.039
MetS					
No	349 (58.6%)	32 (60.4%)	74 (54.8%)	341 (64.5%)	0.101 *
Yes	247 (41.4%)	21 (39.6%)	61 (45.2%)	188 (35.5%)	
Number of retrieved oocytes	18.52 ± 10.46	16.40 ± 7.19	18.59 ± 11.17	16.37 ± 9.06	0.001
Maturation rate (within ICSI, %)	78.99 ± 17.22	72.40 ± 16.12	81.27 ± 17.56	81.49 ± 17.64	0.282
Insemination method					
Conventional IVF	432 (72.5%)	42 (79.2%)	94 (69.6%)	366 (69.2%)	0.608
ICSI	145 (24.3%)	11 (20.8%)	37 (27.4%)	144 (27.2%)	
Half-ICSI	19 (3.2%)	0 (0%)	4 (3.0%)	19 (3.6%)	
Fertilization rate (%)					
IVF	79.3 ± 19.1	79.6 ± 17.0	80.5 ± 17.4	80.3 ± 18.0	0.849
ICSI	78.7 ± 15.8	78.7 ± 14.8	72.6 ± 18.2	75.7 ± 19.8	0.218
2PN rate (%)					
IVF	64.3 ± 19.7	63.5 ± 19.0	62.1 ± 19.0	64.0 ± 20.8	0.807
ICSI	71.3 ± 18.2	68.9 ± 16.9	67.3 ± 17.1	68.2 ± 21.9	0.520
Rate of good quality embryos (%)	74.1 ± 24.8	71.5 ± 22.0	74.3 ± 25.1	73.2 ± 25.7	0.826
Endometrial thickness on the trigger day	9.88 ± 1.76	9.93 ± 1.82	10.01 ± 1.59	10.25 ± 1.68	0.023
Transfer strategy					
Fresh ET	202 (33.9%)	19 (35.8%)	50 (37.0%)	235 (44.4%)	0.004
Frozen ET	394 (66.1%)	34 (64.2%)	85 (63.0%)	294 (55.6%)	
Days of ET in fresh cycles					
D3	198 (98.0%)	19 (100%)	50 (100%)	226 (96.2%)	0.309
D5/6	4 (2.0%)	0 (0%)	0 (0%)	9 (3.8%)	
Number of embryos transferred in fresh cycles					
1	19 (9.4%)	1 (5.3%)	3 (6.0%)	34 (14.5%)	0.162
2	183 (90.6%)	18 (94.7%)	47 (94.0%)	201 (85.5%)	

* The *p* value between phenotype A and phenotype D was 0.043; and the *p* value between phenotype C and phenotype D was 0.041. Abbreviations: PCOS, polycystic ovary syndrome; BMI, body mass index; TT, total testosterone; AND, androstenedione; DHEAS, dehydroepiandrosterone sulfate; SHBG, sex-hormone-binding globulin; FAI, free androgen index; LH, luteinizing hormone; FSH, follicle-stimulating hormone; AMH, anti-Müllerian hormone; IGF-1, insulin-like growth factor 1; HOMA-IR, Homeostasis Model Assessment of Insulin Resistance; MetS, metabolic syndrome; ICSI, intracytoplasmic sperm injection; IVF, in vitro fertilization; PN, pronucleus; ET, embryo transfer.

**Table 2:** Clinical characteristics of phenotypes in PCOS based on androgen level and BMI.

Characteristics	Normal Androgen (*n* = 529)	HA (*n* = 784)	*p* Value	Normal Weight <24 kg/m^2^ (*n* = 580)	Overweight ≥24 kg/m^2^ (*n* = 733)	*p* Value
Age (years)	30.59 ± 3.43	30.18 ± 3.45	0.034	30.30 ± 3.41	30.38 ± 3.48	0.671
BMI (kg/m^2^)	24.86 ± 4.03	25.11 ± 3.96	0.267	21.47 ±1.75	27.82 ± 2.88	<0.001
TT (nmol/L)	1.06 ± 3.63	1.32 ± 0.66	0.048	1.09 ± 0.55	1.31 ± 3.09	0.055
AND (nmol/L)	6.66 ± 2.94	14.85 ± 5.05	<0.001	11.77 ± 6.07	11.73 ± 5.78	0.911
DHEAS (μmol/L)	5.33 ± 1.86	6.97 ± 3.05	<0.001	6.20 ± 2.49	6.50 ± 3.00	0.130
SHBG (nmol/L)	44.83 ± 46.01	33.61 ± 31.73	0.008	51.52 ± 45.91	28.76 ± 28.31	<0.001
FAI	3.29 ± 2.25	6.02 ± 5.10	<0.001	3.56 ± 3.69	6.02 ± 4.74	<0.001
LH/FSH	0.98 ± 0.72	1.06 ± 0.63	0.043	1.13 ± 0.80	0.95 ± 0.54	<0.001
AMH (ng/mL)	7.16 ± 3.64	9.04 ± 4.88	<0.001	9.48 ± 4.88	7.33 ± 3.95	<0.001
IGF-1 (ng/mL)	217.41 ± 67.74	222.97 ± 62.85	0.148	232.62 ± 66.11	211.49 ± 62.43	<0.001
HOMA-IR	2.64 ± 2.78	2.99 ± 2.17	0.011	1.90 ± 1.11	3.61 ± 2.90	<0.001
MetS						
No	341 (64.5%)	455 (58.0%)	0.019	455 (78.4%)	341 (46.5%)	<0.001
Yes	188 (35.5%)	329 (42.0%)		125 (21.6%)	392 (53.5%)	
Number of retrieved oocytes	16.37 ± 9.06	18.39 ± 10.40	<0.001	19.32 ± 9.97	16.20 ± 9.68	<0.001
Maturation rate (within ICSI, %)	81.49 ± 17.64	79.05 ± 17.24	0.204	80.73 ± 16.14	79.59 ± 18.41	0.552
Insemination method						
Conventional IVF	366 (69.2%)	568 (72.4%)	0.420	409 (70.5%)	525 (71.6%)	0.548
ICSI	144 (27.2%)	193 (24.6%)		149 (25.7%)	188 (25.6%)	
Half-ICSI	19 (3.6%)	23 (2.9%)		22 (3.8%)	20 (2.7%)	
Fertilization rate (%)						
IVF	80.3 ± 18.0	79.5 ± 18.7	0.503	80.1 ± 18.7	79.6 ± 18.2	0.672
ICSI	75.7 ± 19.8	77.5 ± 16.3	0.341	76.9 ± 17.5	76.6 ± 18.3	0.879
2PN rate (%)						
IVF	64.0 ± 20.8	63.9 ± 19.5	0.907	65.0 ± 19.3	63.1 ± 20.5	0.147
ICSI	68.2 ± 21.9	70.4 ± 17.9	0.312	69.1 ± 19.3	69.7 ± 20.1	0.783
Rate of good quality embryos (%)	73.2 ± 25.7	74.0 ± 24.6	0.567	72.6 ± 24.3	74.5 ± 25.7	0.166
Endometrial thickness on the trigger day	10.25 ± 1.68	9.90 ± 1.74	0.002	9.96 ± 1.71	10.12 ± 1.73	0.156
Transfer strategy						
Fresh ET	235 (44.4%)	271 (34.6%)	<0.001	170 (29.3%)	336 (45.8%)	<0.001
Frozen ET	294 (55.6%)	513 (65.4%)		410 (70.7%)	397 (54.2%)	
Days of ET in fresh cycles						
D3	226 (96.2%)	267 (98.5%)	0.095	167 (98.2%)	326 (97.0%)	0.416
D5/6	9 (3.8%)	4 (1.5%)		3 (1.8%)	10 (3.0%)	
Number of embryos transferred in fresh cycles						
1	34 (14.5%)	23 (8.5%)	0.034	16 (9.4%)	41 (12.2%)	0.348
2	201 (85.5%)	248 (91.5%)		154 (90.6%)	295 (87.8%)	

Abbreviations: PCOS, polycystic ovary syndrome; BMI, body mass index; HA, hyperandrogenism; TT, total testosterone; AND, androstenedione; DHEAS, dehydroepiandrosterone sulfate; SHBG, sex-hormone-binding globulin; FAI, free androgen index; LH, luteinizing hormone; FSH, follicle-stimulating hormone; AMH, anti-Müllerian hormone; IGF-1, insulin-like growth factor 1; HOMA-IR, Homeostasis Model Assessment of Insulin Resistance; MetS, metabolic syndrome; ICSI, intracytoplasmic sperm injection; IVF, in vitro fertilization; PN, pronucleus; ET, embryo transfer.

**Table 3:** Clinical characteristics of phenotypes in PCOS patients with or without MetS.

Characteristics	No MetS (*n* = 796)	MetS (*n* = 517)	*p* Value
Age (years)	30.22 ± 3.39	30.52 ± 3.54	0.123
BMI (kg/m^2^)	23.85 ± 3.74	26.79 ± 3.69	<0.001
TT (nmol/L)	1.26 ± 2.97	1.15 ± 0.62	0.413
AND (nmol/L)	11.57 ± 5.88	12.01 ± 5.94	0.196
DHEAS (μmol/L)	6.47 ± 2.69	6.22 ± 2.94	0.243
SHBG (nmol/L)	45.45 ± 43.28	25.16 ± 21.85	<0.001
FAI	4.18 ± 3.78	6.56 ± 5.22	<0.001
LH/FSH	1.06 ± 0.63	0.99 ± 0.72	0.086
AMH (ng/mL)	8.60 ± 4.48	7.79 ± 4.53	0.001
IGF-1 (ng/mL)	224.27 ± 64.05	215.21 ± 65.88	0.019
HOMA-IR	2.25 ± 1.42	3.79 ± 3.25	<0.001
PCOS phenotypes			
RC-PCOS			
Phenotype A	349 (43.8%)	247 (47.8%)	0.101
Phenotype B	32 (4.0%)	21 (4.1%)	
Phenotype C	74 (9.3%)	61 (11.8%)	
Phenotype D	341 (42.8%)	188 (36.4%)	
HA-based PCOS			
Normal androgen	341 (42.8%)	188 (36.4%)	0.019
HA	455 (57.2%)	329 (63.6%)	
BMI-based PCOS			
Normal weight	455 (57.2%)	125 (24.2%)	<0.001
Overweight	341 (42.8%)	392 (75.8%)	
Number of retrieved oocytes	18.33 ± 9.62	16.41 ± 10.29	0.001
Maturation rate (within ICSI, %)	80.81 ± 15.74	78.84 ± 20.07	0.321
Insemination method			
Conventional IVF	549 (69.0%)	385 (74.5%)	0.030
ICSI	215 (27.0%)	122 (23.6%)	
Half-ICSI	32 (4.0%)	10 (1.9%)	
Fertilization rate (%)			
IVF	79.9 ± 18.1	79.8 ± 18.8	0.931
ICSI	76.7 ± 17.7	76.7 ± 18.4	0.987
2PN rate (%)			
IVF	64.4 ± 19.8	63.3 ± 20.3	0.432
ICSI	68.5 ± 19.7	71.2 ± 19.6	0.225
Rate of good quality embryos (%)	73.2 ± 24.8	74.3 ± 25.4	0.411
Endometrial thickness on the trigger day	10.03 ± 1.74	10.09 ± 1.68	0.600
Transfer strategy			
Fresh ET	274 (34.4%)	232 (44.9%)	<0.001
Frozen ET	522 (65.6%)	285 (55.1%)	
Days of ET in fresh cycles			
D3	266 (97.1%)	227 (97.8%)	0.588
D5/6	8 (2.9%)	5 (2.2%)	
Number of embryos transferred in fresh cycles			
1	34 (12.4%)	23 (9.9%)	0.376
2	240 (87.6%)	209 (90.1%)	

Abbreviations: PCOS, polycystic ovary syndrome; MetS, metabolic syndrome; BMI, body mass index; TT, total testosterone; AND, androstenedione; DHEAS, dehydroepiandrosterone sulfate; SHBG, sex-hormone-binding globulin; FAI, free androgen index; LH, luteinizing hormone; FSH, follicle-stimulating hormone; AMH, anti-Müllerian hormone; IGF-1, insulin-like growth factor 1; HOMA-IR, Homeostasis Model Assessment of Insulin Resistance; RC, Rotterdam criteria; HA, hyperandrogenism; ICSI, intracytoplasmic sperm injection; IVF, in vitro fertilization; PN, pronucleus; ET, embryo transfer.

**Table 4:** Cumulative pregnancy outcomes of phenotypes in PCOS patients across various classifications (*n* = 1313).

PCOS Phenotypes	Clinical Pregnancy			Live Birth			Preterm Birth			Miscarriage			Twin Pregnancy			GDM			PIH		
	No	Yes	*p* Value	No	Yes	*p* Value	No	Yes	*p* Value	No	Yes	*p* Value	No	Yes	*p* Value	No	Yes	*p* Value	No	Yes	*p* Value
RC-PCOS																					
Phenotype A	194	402	0.686	323	273	0.908	221	52	0.014	273	129	0.674	220	53	0.247	258	15	0.142	265	8	0.274
Phenotype B	19	34		29	24		20	4		24	10		21	3		24	0		23	1	
Phenotype C	51	84		75	60		40	20		60	24		43	17		59	1		60	0	
Phenotype D	180	349		278	251		213	38		251	98		206	45		231	20		248	3	
HA-based PCOS																					
Normal androgen	180	349	0.894	278	251	0.495	213	38	0.056	251	98	0.303	206	45	0.439	231	20	0.073	248	3	0.247
HA	264	520		427	357		281	76		357	163		284	73		341	16		348	9	
BMI-based PCOS																					
Normal weight	161	419	<0.001	271	309	<0.001	257	52	0.217	309	110	0.019	248	61	0.833	293	16	0.430	299	10	0.047
Overweight	283	450		434	299		237	62		299	151		242	57		279	20		297	2	
MetS-based PCOS																					
No MetS	238	558	<0.001	394	402	<0.001	338	64	0.013	402	156	0.074	327	75	0.513	388	14	<0.001	395	7	0.565
MetS	206	311		311	206		156	50		206	105		163	43		184	22		201	5	

Abbreviations: PCOS, polycystic ovary syndrome; RC, Rotterdam criteria; HA, hyperandrogenism; BMI, body mass index; MetS, metabolic syndrome; GDM, gestational diabetes mellitus; PIH, pregnancy-induced hypertension.

**Table 5:** Univariate and multivariate analyses of factors associated with cumulative pregnancy outcomes.

Characteristics	Clinical Pregnancy				Live Birth				Preterm Birth				GDM				PIH			
	Univariate Analysis		Multivariate Analysis		Univariate Analysis		Multivariate Analysis		Univariate Analysis		Multivariate Analysis		Univariate Analysis		Multivariate Analysis		Univariate Analysis		Multivariate Analysis	
	OR (95% CIs)	*p* Value	OR (95% CIs)	*p* Value	OR (95% CIs)	*p* Value	Adjusted OR (95% CIs)	*p* Value	OR (95% CIs)	*p* Value	Adjusted OR (95% CIs)	*p* Value	OR (95% CIs)	*p* Value	Adjusted OR (95% CIs)	*p* Value	OR (95% CIs)	*p* Value	Adjusted OR (95% CIs)	*p* Value
Age (years)	1.005 (0.972, 1.039)	0.763	/	0.317	0.975 (0.945, 1.007)	0.120	/	0.265	0.959 (0.902, 1.020)	0.183	/	0.170	1.072 (0.970, 1.185)	0.175	/	0.352	1.102 (0.930, 1.305)	0.264	/	0.272
Type of infertility																				
Primary	Reference				Reference				Reference				Reference				Reference			
Secondary	0.836 (0.651, 1.074)	0.162	/	0.136	0.912 (0.717, 1.160)	0.454	/	0.399	1.203 (0.771, 1.876)	0.416	/	0.285	0.736 (0.328, 1.648)	0.456	/	0.580	1.897 (0.594, 6.063)	0.280	/	0.394
Infertility duration (years)	0.966 (0.920, 1.014)	0.167	/	0.467	0.969 (0.924, 1.016)	0.189	/	0.916	1.016 (0.934, 1.105)	0.715	/	0.429	1.105 (0.980, 1.246)	0.104	/	0.114	1.209 (1.020, 1.433)	0.029	1.200 (1.005, 1.432)	0.044
BMI (kg/m^2^)	0.973 (0.963, 0.983)	<0.001	0.930 (0.901, 0.960)	<0.001	0.920 (0.895, 0.947)	<0.001	0.918 (0.888, 0.949)	<0.001	1.042 (0.988, 1.098)	0.127	/	0.849	1.076 (0.989, 1.170)	0.089	/	0.828	0.895 (0.757, 1.060)	0.198	/	0.076
LH/FSH	1.190 (0.986, 1.436)	0.069	/	0.544	1.247 (1.049, 1.481)	0.012	/	0.115	0.943 (0.701, 1.269)	0.700	/	0.803	1.358 (0.998, 1.847)	0.052	/	0.085	0.405 (0.111, 1.480)	0.172	/	0.229
AMH (ng/mL)	1.040 (1.013, 1.068)	< 0.001	/	0.182	1.032 (1.007, 1.057)	0.011	/	0.450	0.977 (0.934, 1.022)	0.313	/	0.540	0.949 (0.875, 1.029)	0.207	/	0.103	0.974 (0.855, 1.109)	0.686	/	0.645
HOMA-IR	0.971 (0.927, 1.017)	0.217	/	0.124	0.972 (0.926, 1.021)	0.257	/	0.074	1.046 (0.984, 1.110)	0.148	/	0.415	1.111 (1.021, 1.209)	0.015	/	0.071	1.022 (0.888, 1.177)	0.762	/	0.354
MetS																				
No	Reference				Reference				Reference				Reference				Reference			
Yes	0.644 (0.510, 0.812)	<0.001	/	0.091	0.649 (0.519, 0.812)	<0.001	0.748 (0.581, 0.963)	0.024	1.693 (1.117, 2.565)	0.013	1.655 (1.079, 2.537)	0.021	3.314 (1.658, 6.624)	0.001	2.411 (1.151, 5.048)	0.020	1.404 (0.440, 4.478)	0.567	/	0.697

Abbreviations: OR, odds ratio; CI, confidence interval; BMI, body mass index; LH, luteinizing hormone; FSH, follicle-stimulating hormone; AMH, anti-Müllerian hormone; HOMA-IR, Homeostasis Model Assessment of Insulin Resistance; MetS, metabolic syndrome; GDM, gestational diabetes mellitus; PIH, pregnancy-induced hypertension.

## Data Availability

The data presented in this study are available on request from the corresponding author.

## Supplementary Materials

The following supporting information can be downloaded at: https://www.mdpi.com/article/10.3390/jcm12155073/s1, Table S1: Pregnancy outcomes of PCOS patients with varied phenotypes in fresh transfer cycles. (*n* = 506); Table S2: Univariate and multivariate analyses of factors associated with pregnancy outcomes in fresh transfer cycles.
