# Supplementary material for: Metabolic Syndrome Rather Than Other Phenotypes in PCOS as a Predictive Indicator for Clinical Outcomes in IVF: Comprehensive Phenotypic Assessment across All PCOS Classifications
Source: J Clin Med. 2023 Aug 2;12(15):5073. doi: 10.3390/jcm12155073 (PMC10420246; doi:10.3390/jcm12155073)
Supplement: Supplementary file 1 [file jcm-12-05073-s001.zip › jcm-2448127-supplementary.pdf]

Table S1. Pregnancy outcomes of PCOS patients with varied phenotypes in fresh transfer cycles. (n = 506).

PCOS phenotypes	Clinical Pregnancy			Live Birth			Preterm Birth			Miscarriage			Twin Pregnancy		
	No	Yes	*p* Value	No	Yes	*p* Value	No	Yes	*p* Value	No	Yes	*p* Value	No	Yes	*p* Value
RC-PCOS															
Phenotype A	117	85	0.884	150	52	0.752	40	12	0.930	52	33	0.810	38	14	0.438
Phenotype B	12	7		16	3		2	1		3	4		2	1	
Phenotype C	29	21		38	12		10	2		12	9		6	6	
Phenotype D	130	105		172	63		49	14		63	42		40	23	
HA-based PCOS															
Normal androgen	130	105	0.499	172	63	0.592	49	14	0.982	63	42	0.915	40	23	0.534
HA	158	113		204	67		52	15		67	46		46	21	
BMI-based PCOS															
Normal weight	87	83	0.064	117	53	0.045	42	11	0.724	53	30	0.319	37	16	0.465
Overweight	210	135		259	77		59	18		77	58		49	28	
MetS-based PCOS															
No MetS	138	136	0.001	192	82	0.018	68	14	0.061	82	54	0.798	55	27	0.772
MetS	150	82		184	48		33	15		48	34		31	17	

Abbreviations: PCOS, polycystic ovary syndrome; RC, Rotterdam criteria; HA, hyperandrogenism; BMI, body mass index; MetS, metabolic syndrome.

Table S2. Univariate and multivariate analyses of factors associated with pregnancy outcomes in fresh transfer cycles.

Characteristics	Clinical Pregnancy				Live Birth			
	Univariate Analysis		Multivariate Analysis		Univariate Analysis		Multivariate Analysis	
	OR (95% CIs)	*p* Value	Adjusted OR (95% CIs)	*p* Value	OR (95% CIs)	*p* Value	Adjusted OR (95% CIs)	*p* Value
Age (years)	0.980 (0.929, 1.033)	0.443	/	0.306	0.953 (0.897, 1.012)	0.113	/	0.061
Type of infertility								
Primary	Reference				Reference			
Secondary	0.773 (0.522, 1.145)	0.199	/	0.174	0.477 (0.291, 0.781)	0.003	0.534 (0.322, 0.886)	0.015
Infertility duration (years)	0.968 (0.897, 1.045)	0.408	/	0.996	0.965 (0.884, 1.054)	0.430	/	0.750
BMI (kg/m^2)	0.965 (0.924, 1.008)	0.110	/	0.160	0.934 (0.888, 0.983)	0.009	0.928 (0.877, 0.982)	0.009
LH/FSH	0.885 (0.639, 1.226)	0.463	/	0.529	0.831 (0.563, 1.227)	0.353	/	0.350
AMH (ng/mL)	0.953 (0.907, 1.002)	0.060	0.940 (0.893, 0.989)	0.018	0.953 (0.900, 1.010)	0.106	0.914 (0.857, 0.974)	0.006
HOMA-IR	1.015 (0.954, 1.081)	0.630	/	0.210	1.003 (0.937, 1.075)	0.921	/	0.191
MetS								
No	Reference				Reference			
Yes	0.555 (0.388, 0.794)	0.001	0.526 (0.364, 0.760)	0.001	0.611 (0.406, 0.920)	0.018	/	0.095

Continued

Characteristics	Preterm Birth			
	Univariate Analysis		Multivariate Analysis	
	**OR (95% CIs)**	***p* Value**	**Adjusted OR (95% CIs)**	***p* Value**
Age (years)	0.964 (0.853, 1.088)	0.549	/	0.335
Type of infertility				
Primary	Reference			
Secondary	2.024 (0.764, 5.359)	0.156	/	0.233
Infertility duration (years)	1.008 (0.858, 1.185)	0.919	/	0.822
BMI (kg/m^2)	1.021 (0.924, 1.127)	0.686	/	0.954
LH/FSH	1.686 (0.687, 4.136)	0.254	/	0.265
AMH (ng/mL)	0.983 (0.861, 1,121)	0.793	/	0.864
HOMA-IR	0.983 (0.878, 1.101)	0.772	/	0.442
MetS				
No	Reference			
Yes	2.208 (0.954, 5.107)	0.064	2.378 (1.015, 5.569)	0.046

Abbreviations: OR, odds ratio; CI, confidence interval; BMI, body mass index; LH, luteinizing hormone; FSH, follicle-stimulating hormone; AMH, anti-Müllerian hormone; HOMA-IR, Homeostasis Model Assessment of Insulin Resistance; MetS, metabolic syndrome.
